# Laser-Assisted Gingivectomy in Amlodipine-Induced Gingival Enlargement: A Case Report

**DOI:** 10.7759/cureus.75761

**Published:** 2024-12-15

**Authors:** Rasica Kaliyaperumal, Kumar Appusamy, Sabitha Gokulraj, Karthik Rajaram Mohan, Subhalakshmi Venkatraman

**Affiliations:** 1 Oral Medicine and Radiology, Vinayaka Mission's Sankarachariyar Dental College, Vinayaka Mission's Research Foundation (Deemed to be University), Salem, IND; 2 Oral Pathology and Microbiology, Vinayaka Mission's Sankarachariyar Dental College, Vinayaka Mission's Research Foundation (Deemed to be University), Salem, IND

**Keywords:** amlodipine, bone loss, gingival enlargement, gingivectomy, laser

## Abstract

Gingival enlargements are mostly plaque-induced. Other than plaque, a few genetic conditions also cause enlargements of the gingiva. In recent years, there has been a notable rise in drug-induced gingival overgrowth (DIGO) linked to the increased use of medications for various systemic conditions. Commonly implicated drug classes include antihypertensives, anticonvulsants, immunosuppressants, and calcium channel blockers. These medications can lead to gingival overgrowth, which adversely affects oral function, esthetics, and hygiene. In this paper, we report the case of a 47-year-old male patient with hypertension who experienced amlodipine-induced gingival enlargement. In this case report, laser was also incorporated into the treatment plan for the reduction of gingival growth.

## Introduction

Drug-induced gingival enlargement is a condition where certain medications cause an overgrowth of gingival tissue. Commonly implicated drugs include calcium channel blockers, immunosuppressants, and anticonvulsants, each affecting gingival tissue by triggering excessive collagen production and cell growth [1,2]. Amlodipine-induced gingival enlargement is an uncommon but significant side effect of amlodipine, a calcium channel blocker widely prescribed to manage hypertension and angina [3]. Though typically reversible upon discontinuation or adjustment of the medication, untreated cases can result in complications such as inflammation, bleeding, and difficulty with oral hygiene, and in most proliferated instances, surgical intervention is recommended [4].

## Case presentation

**Figure 1:**
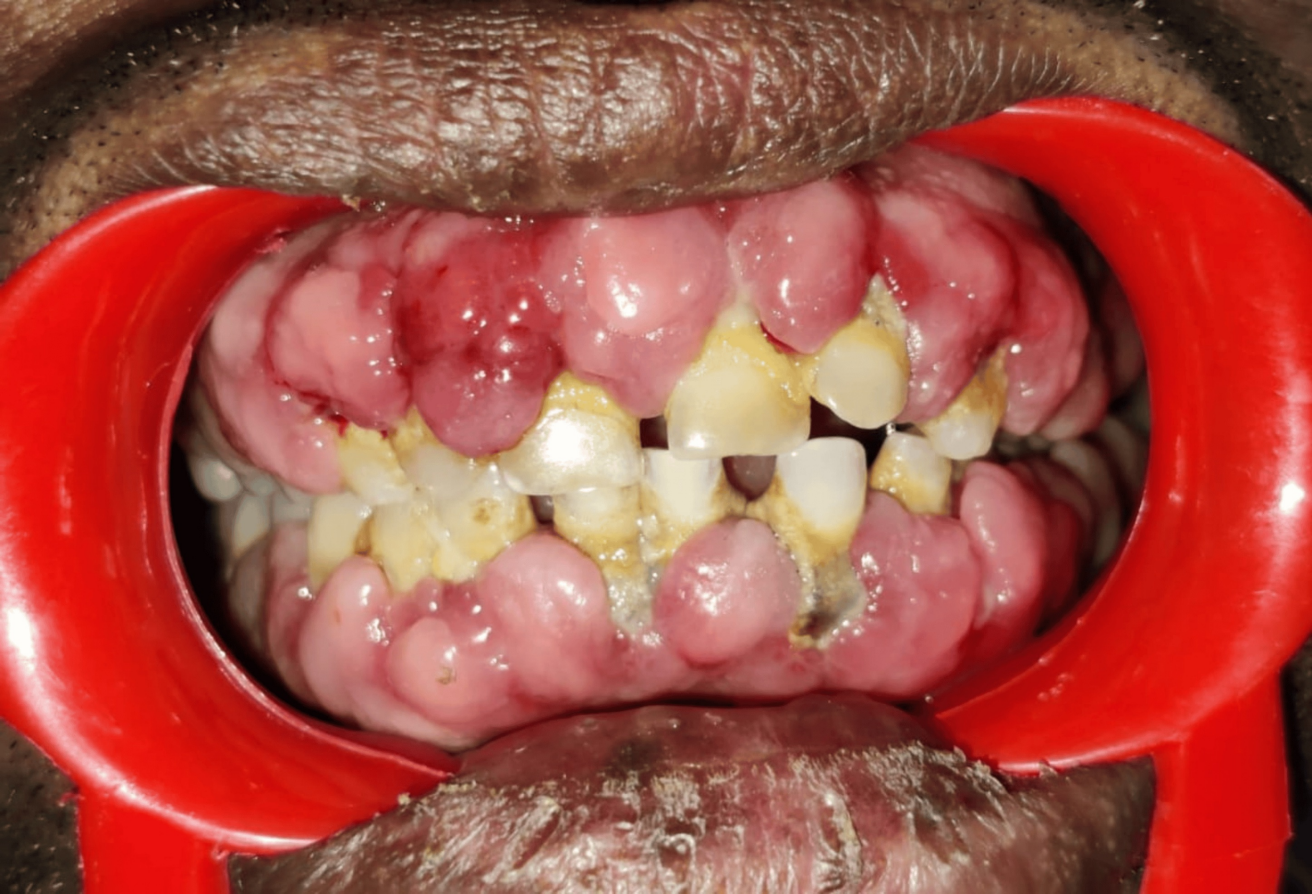
A clinical photograph showing a view of gingival enlargement

A 47-year-old male patient presented to our outpatient department with a chief complaint of enlargement of his gums for the past year. During the intraoral examination, it was noted that there was an overgrowth of overlying soft tissue on the maxillary and mandibular arches (Figure 1).

**Figure 2:**
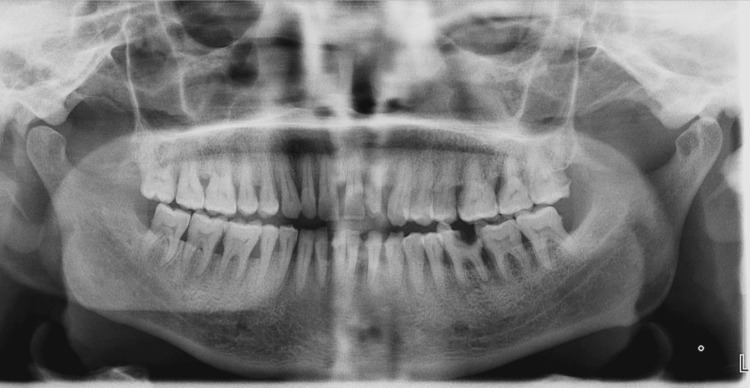
A panoramic radiograph of the patient showing multiple areas of vertical and horizontal bone loss

The growth was painless, nodular, and erythematous in appearance, and bleeding on probing was seen. A detailed case history revealed that the patient had previously visited a government hospital with the same illness, and medication was prescribed. The patient was a known diabetic for the past seven years, had hypertension for the past four years, and was under medication for both. For hypertension, the patient was taking amlodipine 5 mg twice daily. Orthopantomography showed generalized interdental bone loss (Figure 2).

Based on the clinical and drug history of the patient, a provisional diagnosis of amlodipine-induced gingival enlargement was made. For consideration of switching the drug (amlodipine) with another antihypertensive drug, the patient was referred to the physician. He was treated with a combination of beta (β)-blockers and angiotensin-converting enzyme (ACE) inhibitors.

The patient was advised to maintain good oral hygiene, given a chlorhexidine mouth rinse, and recalled two weeks after the inflammation subsided. 

**Figure 3:**
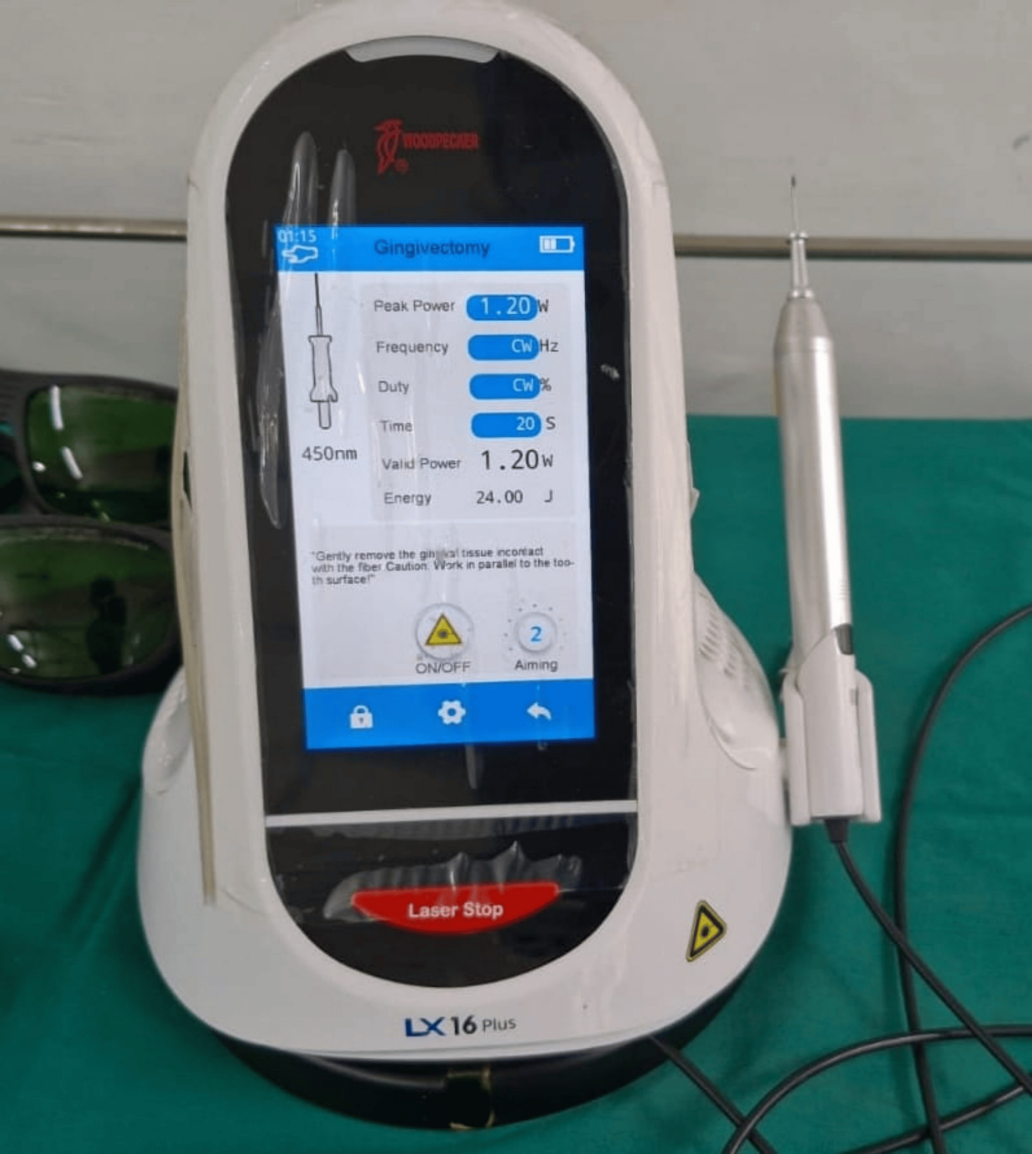
The Woodpecker LX16 Plus laser device This device operates at a wavelength of 450 nm, peak power of 1.2 watts, valid power of 1.2 watts, time of 20 seconds, energy of 24 Jules, and in pulse mode. It is designed for precise soft tissue gingivectomy procedures.

A gingivectomy was performed under local anesthesia in the maxillary first quadrant to remove the fibrous tissue surgically. After one week of gingivectomy, the patient was recalled and asked to report for a laser-assisted gingivectomy in other quadrants with the parameters of the Woodpecker LX16 Plus laser device (Guilin Woodpecker Medical Instrument Co., Ltd, Guilin, China) operating at a wavelength of 450 nm, peak power of 1.2 watts, valid power of 1.2 watts, time of 20 seconds, energy of 24 Jules, and in pulse mode. It is designed for precise soft tissue gingivectomy procedures (Figure 3).

**Figure 4:**
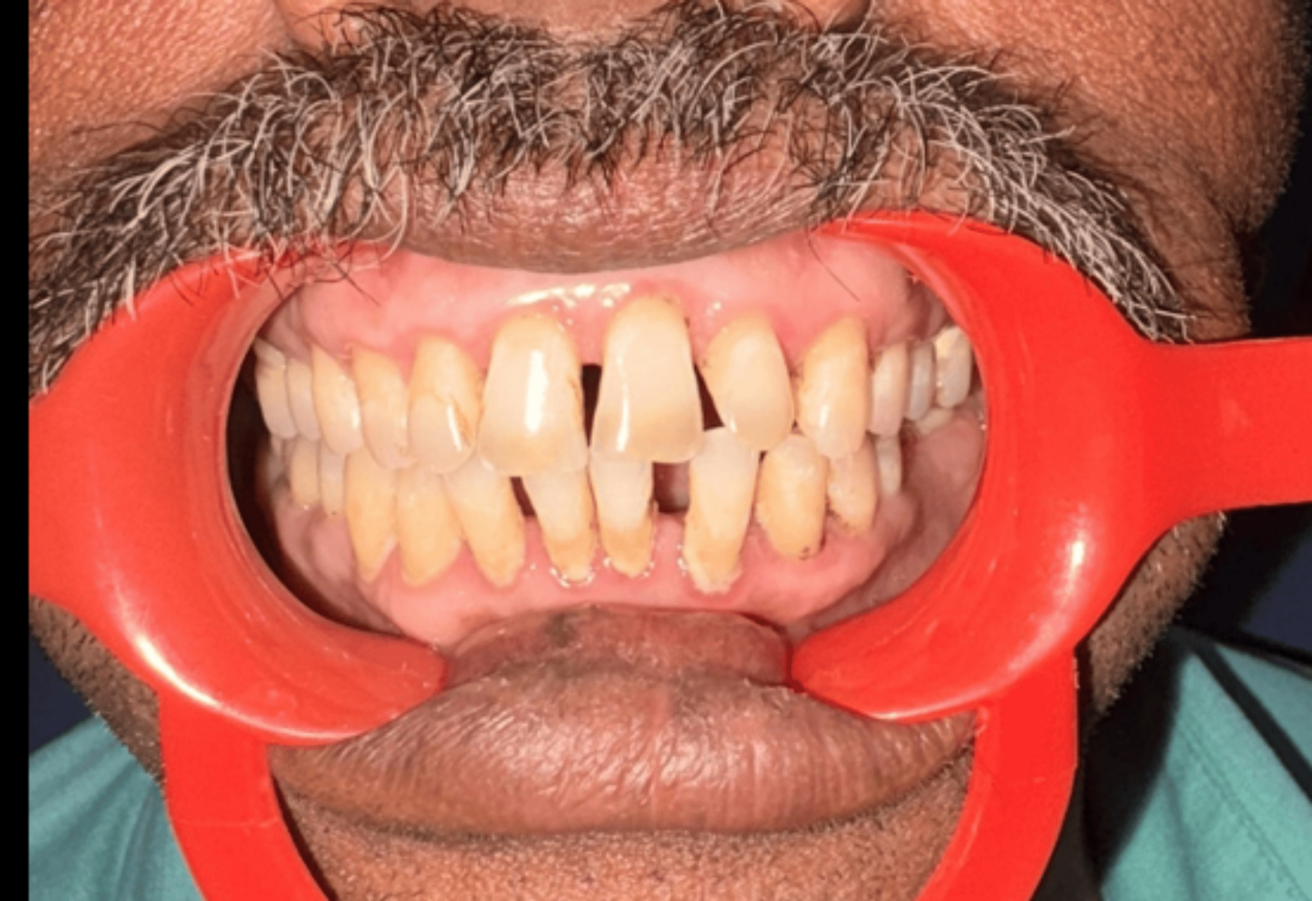
A clinical photograph showing the patient's postoperative picture

Post gingivectomy, the patient's gingival status improved in both conventional and laser methods (Figure 4).

## Discussion

Drug-induced gingival enlargement, also known as drug-induced gingival overgrowth (DIGO) or drug-induced gingival hyperplasia, is a condition where the gums become swollen and enlarged due to certain medications [5]. The accumulation of extracellular matrix and fibrosis act as fundamental etiological mechanisms. In patients with amlodipine-induced gingival enlargement, the elevated expression of TGF-β1 and CTGF, which are key regulators of connective tissue growth and repair, plays a significant role in the pathogenesis of the condition [2]. Around 20 medications have been identified as potential causes of gingival overgrowth [3-5]. This enlargement can affect one or more gum areas, leading to esthetic concerns and sometimes causing difficulty eating and speaking. Several medication classes are known to potentially cause gingival enlargement, including anticonvulsants, calcium channel blockers, immunosuppressants, and more [6, 7].

Amlodipine, a more recent long-acting dihydropyridine that belongs to calcium channel blockers, is commonly used to treat hypertension and certain other heart conditions [8]. Its mechanism of action involves its effects on calcium channels in cardiac and vascular smooth muscle cells [8]. The major disadvantage of the drug is it causes the gingival overgrowth. The exact mechanism of amlodipine-induced gingival enlargement must still be fully understood. However, it is thought that it involves changes in connective tissue metabolism within gums [8]. The first reported side effect of causing gingival enlargement by amlodipine was by Seymour et al. in 1994 [9]. The prevalence of amlodipine-induced gingival enlargement is considered relatively rare compared to other drug-induced gingival enlargements [10]. Poor oral hygiene and existing periodontal disease can exacerbate the severity of gingival enlargement in individuals taking amlodipine [10]. Gingival enlargement usually appears in the first three months of taking amlodipine therapy. The enlargement tends to be painless and symmetric and most commonly affects the anterior teeth' interdental papillae and marginal gingiva [10]. Patients may experience swollen and enlarged gums covering the teeth partially or entirely [11]. This enlargement can be painless but may cause aesthetic concerns and difficulties with oral hygiene and clinical symptoms, including bleeding and speech disturbances [11]. Not every patient taking amlodipine will develop gingival enlargement. The incidence of amlodipine-induced gingival enlargement was reported to be much lower than that of nifedipine, another calcium channel blocker [12].

Clinically, amlodipine-induced gingival enlargement presents as firm, painless, and symmetrical overgrowth. The enlargement may start shortly after amlodipine therapy or develop gradually over time. The risk factors include higher doses of amlodipine, longer duration of treatment, and individual susceptibility [12].

Managing amlodipine-induced gingival enlargement involves a combination of preventive measures, regular monitoring, and sometimes therapeutic interventions. Consult with the prescribing physician to discuss whether reducing the dosage or switching to an alternative medication is clinically feasible or appropriate; consider switching to an alternative antihypertensive medication that does not have a significant risk of causing gingival enlargement [12].

The standard non-surgical treatment for drug-induced gingival enlargement is professional debridement with scaling and root planning to give some relief [13]. In cases where conservative measures fail to resolve the gingival overgrowth or when the enlargement is severe, surgical removal of the excess gum tissue (gingivectomy) may be necessary [13].

The surgical treatment of drug-induced gingival enlargement was advocated by Thompson and Gillespie in 1941 [14]. It is often considered in cases of severe gingival enlargement that do not respond adequately to conservative measures such as improved oral hygiene and medication adjustments. Gingivectomy is an effective form of treatment, and it is indicated as technically simple, precise, and less damaging to the surrounding tissue. Perioperative bleeding is the primary drawback of surgical excision. Diode, argon, and carbon dioxide lasers can be used as alternatives to surgical excision [14].

Laser-assisted gingivectomy has emerged as an alternative to traditional surgical methods for treating gingival enlargement, including cases induced by medication like amlodipine [14]. It offers several advantages for treating gingival enlargement, including precision, reduced discomfort, and faster healing times. In some cases, gingival enlargement may recur, necessitating ongoing monitoring and management [14]. Complications associated with this condition are pain and discomfort, difficulty with oral hygiene, increased risk of periodontal disease, and potential tooth damage [14].

When diagnosing amlodipine-induced gingival enlargement, it's essential to consider and rule out other possible causes of gingival overgrowth. The differential diagnosis includes other DIGO, like phenytoin and cyclosporine; other calcium channel blockers like nifedipine and verapamil; inflammatory gingival enlargements like chronic gingivitis and periodontitis; hormonal causes like puberty, pregnancy, and hormonal imbalances like hyperthyroidism; systemic diseases like leukemia and sarcoidosis; genetic conditions like hereditary gingival fibromatosis; and nutritional deficiencies like vitamin C deficiency (scurvy), which can cause swollen, bleeding gums, and other conditions like fibromas and amyloidosis.

The prognosis for amlodipine-induced gingival enlargement is generally favorable, particularly when the condition is recognized early and managed appropriately. In many cases, gingival enlargement due to amlodipine is reversible once the causative medication is discontinued or replaced and with proper oral care. Ongoing monitoring and maintenance of oral hygiene are crucial to prevent recurrence.

## Conclusions

Amlodipine-induced gingival enlargement, though uncommon, can significantly impact oral health and quality of life if left unaddressed. The condition highlights the importance of recognizing medication side effects and the role of healthcare providers in educating patients about potential oral changes. Effective management strategies include improving oral hygiene practices, regular dental visits, and possibly adjusting or switching medications under a physician's guidance.

Gingival enlargement is often reversible with timely intervention, and symptoms can be minimized or resolved. Raising awareness about this side effect can help patients and providers work together to achieve better overall health outcomes.
